# Use of Artificial Intelligence-Assisted Conversational Agents to Improve Patient Experience Related to Physicians: Cross-Sectional Study in China

**DOI:** 10.2196/76540

**Published:** 2025-10-17

**Authors:** Dehe Li, Heman Zhang, Chuntao Lu, Chunxia Miao

**Affiliations:** 1 School of Management, Xuzhou Medical University, No. 209, Tongshan Road, Xuzhou, 221004, China, 86 15366760560; 2 Jingmen People’s Hospital, Jingmen, China

**Keywords:** conversational agent, artificial intelligence, outpatient services, satisfaction, quantitative, cross-sectional, questionnaire, China

## Abstract

**Background:**

Artificial intelligence-assisted conversational agents have been applied and developed in outpatient departments to improve health services in China. However, there has been little research that evaluates the effect of artificial intelligence-assisted conversational agents on the patient experience related to physicians during outpatient visits.

**Objective:**

This study aimed to examine whether the use of artificial intelligence-assisted conversational agents improves the patient experience related to physicians during outpatient visits and to further find out the difference in the patient experience between conversational agent users and nonusers.

**Methods:**

We used the Chinese Outpatient Experience Questionnaire to survey the patient experience related to physicians during outpatient visits. A sample of 394 adult residents who sought outpatient services from tertiary public hospitals in China was selected by random sampling. The *t* tests were used to test the mean difference in the patient experience scores between conversational agent users and nonusers. Multiple linear regression analysis was further performed to determine whether the use of artificial intelligence-assisted conversational agents during outpatient visits was associated with a better patient experience related to physicians.

**Results:**

Conversational agent users reported significantly higher scores than nonusers in the total patient experience scores (*t*_392_=5.589, *P*<.001), the 19 items and 4 dimensions of physician-patient communication (*t*_392_=5.013, *P*=.006), health information (*t*_392_=5.758, *P*<.001), short-term outcome (*t*_392_=4.608, *P*<.001) and general satisfaction (*t*_392_=5.080, *P*<.001). Multiple linear regression results further showed that after controlling for other factors on participant characteristics, the use of artificial intelligence-assisted conversational agents during outpatient visits significantly influenced the total patient experience scores related to physicians (*B*=0.298, *P*=.01). And the use of artificial intelligence-assisted conversational agents averagely increased the total patient experience scores related to physicians during outpatient visits by 7.51% (0.298/3.97).

**Conclusions:**

The use of artificial intelligence-assisted conversational agents could improve the patient experience related to physicians during outpatient visits, especially in terms of making physician-patient communication better, accessing more targeted health information, ameliorating short-term outcomes, and increasing general satisfaction. Therefore, we suggest that public hospitals should consider the benefits of the artificial intelligence-assisted conversational agents and actively deploy the conversational agents in outpatient departments so as to continuously improve the patient experience related to physicians during outpatient visits.

## Introduction

### Background

Patient experience is the general satisfaction a patient obtains during the process of receiving health care services [1]. In particular, patient experience has been considered as one of the central pillars of health care quality [2,3]. There are currently various developed questionnaires or scales to measure the patient experience in different health care settings, such as the Outpatient Experiences Questionnaire [4,5], the Chinese Patient Experience Questionnaire [6] and the Picker scale [7]. Previous research pointed out that patient experience was closely related to the quality of health care delivery, involving outcomes such as patient safety and clinical effectiveness [3,8]. Some qualitative studies further indicated that a more positive patients’ communication experience with their physicians is related to higher general satisfaction with the quality of health care delivery [9]. Thus, policymakers worldwide increasingly prefer using patient-experience data over performance indicators to evaluate the quality of health care services.

In recent years, the Chinese government has been dedicated to improving the patient experience [10] and encouraged the development and application of artificial intelligence in the health services in various scenarios (eg, conversational agents [11], artificial intelligence-assisted diagnosis [12] and decision-making [13]). This initiative promotes the construction of smart hospitals with the aim of solving the urgent and difficult problems when people seek health services, thereby continuously improving the quality of health services.

Conversational agents are artificial intelligence programs (ie, chatbots) that engage in dialogs with patients via the mobile devices before a consultation with their visit physicians in outpatient departments [14]. With the contextual question-answering agents, the patients’ information on their conditions, symptoms, and past medical history (eg, disease history, examination or medication or operation history, allergy history, family history, and personal history of drinking or smoking) can be collected and then sent to their physicians’ workstations in a structured form [11]. Some review studies have pointed out that artificial intelligence-assisted conversational agents had the potential to save time by reducing the time required for history taking and improve consultation efficiency, thereby resulting in high levels of satisfaction [15,16]. And these studies further indicated there were few quantitative studies that evaluate the above-mentioned effects or outcomes of conversational agents with objective measures [15,16].

### Objectives

Under the national policies on the digital transformation of the health care industry, artificial intelligence-assisted conversational agents have begun applying to enhance the health care delivery in tertiary public hospitals in economically developed regions of China (eg, Shanghai in 2021) [17]. The artificial intelligence-assisted conversational agents have not only helped patients report their information in detail with enough time, but also made their visit physicians quickly grasp their conditions before a consultation [11]. It is apparent that the artificial intelligence-assisted conversational agents play a positive role in improving the efficiency and quality of health care delivery related to physicians.

Despite the application of artificial intelligence-assisted conversational agents in the tertiary public hospitals in economically developed regions of China, evidence to assess their effectiveness in improving health care delivery is lacking to date. Some studies investigated the factors influencing the adoption and continuance intention of patients toward artificial intelligence-assisted conversational agents in outpatient departments [11]. Several studies explored the design of the intelligence-assisted conversational agents [18] and what issues and barriers exist in their usage [19]. Another study assessed the impact of artificial intelligence-based conversational agent on the operational performance [20]. However, there has been little further research that evaluates the effect of artificial intelligence-assisted conversational agents on the patient experience related to physicians during outpatient visits.

Therefore, this study aimed to examine whether the use of artificial intelligence-assisted conversational agents during outpatient visits improves the patient experience related to physicians and to further find out the difference in the patient experience between conversational agent users and nonusers.

## Methods

### Questionnaire Design

The Chinese Outpatient Experience Questionnaire was the basis of our survey, including 6 dimensions (physical environment and convenience, medical service fees, physician-patient communication, health information, short-term outcome, and general satisfaction), 28 items and participant characteristics (eg, sex, age, marital status, education, living place, monthly income, self-rated health status, and visit information) [21]. This outpatient experience questionnaire was verified with good reliability and validity (*χ*^2^/df =2.775, goodness-of-fit index=0.893, comparative fit index=0.930, Tucker-Lewis index=0.921, root mean square error of approximation=0.055, root mean square residual=0.038) [21]. However, we selected the above 4 dimensions (physician-patient communication, health information, short-term outcome, and general satisfaction; Cronbach alpha=.968 in this study) and the corresponding 19 items to survey the outpatient experience related to physicians. Moreover, we also added another question in the section of participant characteristics—“Did you use the artificial intelligence-assisted conversational agents during this outpatient visit?”—to divide the conversational agent users and nonusers.

### Data Collection

The target population was adult residents who sought outpatient services from tertiary public hospitals within the past 2 weeks in China, selected using the random sampling. We used a professional data collection platform (Credamo) in China to create an electronic questionnaire in which to survey the targeted residents. The sample database of the Credamo included more than 3.0 million members with confirmed personal information from all provinces and regions in China [22]. With the support of Credamo, this study distributed electronic questionnaires to the targeted population nationwide and invited them to participate in the survey from April 1 to 15, 2025. Specifically, this study randomly sent the questionnaire links to the members who met the inclusion criteria nationwide through the Credamo in a targeted manner. These criteria were mainly set using the sample feature screening function of the Credamo as follows: being aged 18 years or older, being located within China, and having an outpatient experience in a tertiary public hospital within the past 2 weeks. Each invited participant could click on the link via their mobile phones to access and complete the electronic questionnaire. Before the survey, we introduced the nature and objective of the study and guaranteed that the collected data would not be used for other purposes. The survey was conducted accordingly after an individual’s consent was obtained. Each invited participant was prompted to fill in the electronic questionnaire based on their outpatient experience in tertiary public hospitals within the past 2 weeks. Each internet protocol address could be set to fill in the questionnaire only once.

### Ethical Considerations

The institutional review board of Xuzhou Medical University approved this study before data collection (number 2024Z048). The general information about the nature and objective of this study was also provided and informed at the beginning of the survey as a means of informed consent. All the participants were informed that their participation was voluntary and they were free to refuse or discontinue their participation at any time. And only after an individual’s consent was obtained online could she or he continue to participate in this survey. During the data collection, no identifying information was collected, and the researchers only had access to the user ID. We also provided the participants who met the inclusion criteria and carefully completed the questionnaire with a monetary reward (US $0.70). This online survey was designed in accordance with the CHERRIES checklist.

### Measures

The dependent variable was the total patient experience scores related to physicians in the multiple linear regression analysis. The four dimensions (physician-patient communication, health information, short-term outcome, and general satisfaction) and the corresponding 19 items of the Chinese Outpatient Experience Questionnaire were used to calculate the patient experience scores related to physicians during outpatient visits [21]. Each item was rated on a 5-point Likert scale, with a higher score indicating a better experience [21]. Each dimension score was calculated by adding up the scores of all items in the dimension and then dividing that sum by the total number of items in that dimension. We further calculated the total patient experience scores by summing the scores of the 19 items in the questionnaire and then dividing that sum by the total number of items [1]. Therefore, the total patient experience scores related to physicians ranged from 1 to 5. The independent variables included whether the artificial intelligence-assisted conversational agents were used during this outpatient visit (coded as 1=yes, 0=no), as well as the participant characteristics, including demographic and visit information.

### Statistical Analysis

Descriptive statistics were performed to summarize data on the characteristics of participants. The *t* tests were then used to test the mean difference in the patient experience scores between conversational agent users and nonusers when the data followed a normal distribution. And multiple linear regression analysis was further performed to determine whether the use of artificial intelligence-assisted conversational agents during outpatient visits was associated with a better patient experience related to physicians. Moreover, the average percentage change in the dependent variable associated with a one-unit increase in an independent variable was calculated by dividing the independent variable’s unstandardized regression coefficient value by the mean value of the dependent variable and then multiplying by 100%. Benjamini-Hochberg adjusted *P* values ≤.05 were considered statistically significant. All data analyses were done using SPSS (version 23.0; IBM Corp) and STATA (version 15.0).

## Results

### Participant Characteristics of Conversational Agent Users and Nonusers

A total of 462 online responses were received, and 394 eligible responses remained, whereas 68 responses were excluded because they showed a certain logical contradiction based on the screening question (ie, whether you had an outpatient experience in a tertiary public hospital within the past 2 wk), or they contained the same answers to all questions, or because the time they were filled in was less than 120 seconds. The detailed characteristics of the participants are shown in Table 1. Among these participants, 53.0% (209/394) reported they used conversational agents during this outpatient visit. And the conversational agent users and nonusers differed in the sex (*χ*^2^_1_=9.90, *P*=.002), educational level ( *χ*^2^_2_=6.025, *P*=.049), monthly income (*χ*^2^_3_=24.262, *P*<.001), self-rated health status ( *χ*^2^_2_=31.247, *P*<.001) and physician title (*χ*^2^_3_=9.643, *P*=.02). Moreover, these participants who rated their health status better were more likely to use the conversational agents during this outpatient visit.

**Table 1.** Differences in the participant characteristics of conversational agent users and nonusers.

Characteristic	Overall, n (%)	Conversational agent users, n (%)	Nonusers, n (%)	*χ*^2^ (*df*)	*P* value
Sex				9.90 (1)	.002^a^
Male	120 (30.5)	78 (37.3)	42 (22.7)		
Female	274 (69.5)	131 (62.7)	143 (77.3)		
Age (years)				3.393 (3)	.34
18‐25	187 (47.5)	91 (43.5)	96 (51.9)		
26‐30	82 (20.8)	46 (22.0)	36 (19.5)		
31‐40	80 (20.3)	44 (21.1)	36 (19.5)		
40	45 (11.4)	28 (13.4)	17 (9.2)		
Marital status				2.924 (1)	.09
Unmarried	251 (63.7)	125 (59.8)	126 (68.1)		
Married	143 (36.3)	84 (40.2)	59 (31.9)		
Educational level				6.025 (2)	.049^a^
High School and below	59 (15.0)	30 (14.4)	29 (15.7)		
College and undergraduate	272 (69.0)	154 (73.7)	118 (63.8)		
Postgraduate and above	63 (16.0)	25 (12.0)	38 (20.5)		
Monthly income (US $)				24.262 (3)	<.001^a^
<417.97	139 (35.3)	54 (25.8)	85 (45.9)		
417.97‐696.48	86 (21.8)	58 (27.8)	28 (15.1)		
696.62‐1114.45	82 (20.8)	54 (25.8)	28 (15.1)		
≥1114.59	87 (22.1)	43 (20.6)	44 (23.8)		
Current living place				0.279(1)	.60
Urban areas	315 (79.9)	165 (78.9)	150 (81.1)		
Rural areas	79 (20.1)	44 (21.1)	35 (18.9)		
Self-rated health status				31.247 (2)	<.001^a^
Fair	112 (28.4)	36 (17.2)	76 (41.1)		
Good	198 (50.3)	114 (54.5)	84 (45.4)		
Very good	84 (21.3)	59 (28.2)	25 (13.5)		
Specialty services				7.777(8)	.46
Internal medicine	131 (33.2)	69 (33.0)	62 (33.5)		
Surgery	67 (17.0)	40 (19.1)	27 (14.6)		
Obstetrics and gynecology	33 (8.4)	11 (5.3)	22 (11.9)		
Orthopedics	29 (7.4)	14 (6.7)	15 (8.1)		
Traditional Chinese medicine	24 (6.1)	13 (6.2)	11 (5.9)		
Otorhinolaryngology	23 (5.8)	14 (6.7)	9 (4.9)		
Ophthalmology	17 (4.3)	9 (4.3)	8 (4.3)		
Stomatology	30 (7.6)	18 (8.6)	12 (6.5)		
Dermatology	40 (10.2)	21 (10.0)	19 (10.3)		
Physician title				9.643 (3)	.02^a^
Senior	140 (35.5)	86 (41.1)	54 (29.2)		
Deputy Senior	113 (28.7)	53 (25.4)	60 (32.4)		
Intermediate	119 (30.2)	63 (30.1)	56 (30.3)		
Junior	22 (5.6)	7 (3.3)	15 (8.1)		
Whether this outpatient visit was a revisit				0.126 (1)	.72
Yes	57 (14.5)	29 (13.9)	28 (15.1)		
No	337 (85.5)	180 (86.1)	157 (84.9)		

a Represents a significant difference between the 2 groups.

### Differences in Patient Experience Related to Physicians Between Conversational Agent Users and Nonusers

Table 2 shows the patient experience scores of conversational agent users and nonusers. And the analysis results further showed that there was a significant difference in the total patient experience scores, the 4 dimensions, and the 19 items between the 2 groups.

Specifically, in the total patient experience scores related to physicians, conversational agent users obtained significantly higher scores than nonusers (*t*_392_=5.589, *P*<.001). In these dimensions of physician-patient communication (*t*_392_=5.013, *P*=.006), health information (*t*_392_=5.758, *P*<.001), short-term outcome (*t*_392_=4.608, *P*<.001) and general satisfaction (*t*_392_=5.080, *P*<.001), conversational agent users reported significantly higher scores than nonusers as well.

Moreover, conversational agent users also reported significantly higher scores than nonusers in the 19 items of patient experience related to physicians (see Table 2).

**Table 2.** Patient experience scores of conversational agent users and nonusers.

Dimension/item	Conversational agent users scores, mean (SD)	Nonusers scores, mean (SD)	*t* test (*df*)	*P* value
Physician-patient communication	4.11 (0.74)	3.75 (0.70)	5.013 (392)	.006^a^^,b^
Clear explanation	4.13 (0.78)	3.91 (0.77)	2.753 (392)	<.001^a^
Careful listening	4.22 (0.84)	3.89 (0.81)	3.920 (392)	<.001^a^
Enough time for communication	3.93 (1.00)	3.55 (1.02)	3.726 (392)	<.001^a^
Courtesy and respect attitude	4.20 (0.79)	3.84 (0.81)	4.498 (392)	<.001^a^
Cared about anxieties or fears	4.03 (0.92)	3.52 (1.05)	5.141 (392)	<.001^a^
Involve in decision making	4.04 (0.92)	3.67 (0.99)	3.807 (392)	<.001^a^
Respect opinions	4.10 (0.82)	3.72 (0.85)	4.519 (392)	<.001^a^
Protect personal privacy	4.22 (0.93)	3.87 (0.88)	3.892 (392)	<.001^a^
Health information	4.17 (0.74)	3.73 (0.75)	5.758 (392)	<.001^a^
Explanations for your illness	4.13 (0.91)	3.84 (0.84)	3.324 (392)	.001^a^
Dangerous signals at home	4.22 (0.85)	3.90 (0.83)	3.857 (392)	<.001^a^
Health knowledge	4.13 (0.88)	3.71 (0.99)	4.494 (392)	<.001^a^
Explain following examination	4.17 (0.91)	3.64 (0.96)	5.608 (392)	<.001^a^
Explain examination result	4.16 (0.92)	3.71 (0.97)	4.734 (392)	<.001^a^
Explain drug effects in a way you could understand	4.07 (0.89)	3.54 (1.01)	5.511 (392)	<.001^a^
Medication precautions	4.27 (0.72)	3.79 (0.93)	5.626 (392)	<.001^a^
Short-term outcome	4.19 (0.79)	3.81 (0.84)	4.608 (392)	<.001^a^^,b^
Reduce/prevent from health problems	4.23 (0.84)	3.86 (0.91)	4.155 (392)	<.001^a^
Handle health problems after visit	4.15 (0.86)	3.76 (0.90)	4.401 (392)	<.001^a^
General satisfaction	4.24 (0.76)	3.85 (0.78)	5.080 (392)	<.001^a^^,b^
Satisfaction overall	4.27 (0.81)	3.84 (0.82)	5.210 (392)	<.001^a^
Choose this hospital again	4.22 (0.81)	3.85 (0.86)	4.293 (392)	<.001^a^
Total patient experience scores	4.15 (0.71)	3.76 (0.69)	5.589 (392)	<.001^a^

a Represents a significant difference between the 2 groups.

b Represents the dimensions in the questionnaire.

### Influence of Conversational Agents on Patient Experience Related to Physicians

As shown in Table 3, after controlling for other factors on participant characteristics including demographic and visit information and adjusting the *P* value using Benjamini-Hochberg procedure, whether the conversational agent was used or not during this outpatient visit was a significant factor influencing the total patient experience scores related to physicians (*B*=0.298, *P*=.013). The standardized regression coefficient of whether the conversational agent was used was 0.205. Thus, when other covariates were held constant, the use of the artificial intelligence-assisted conversational agents averagely increased the total patient experience scores related to physicians by 7.51% (0.298/3.97*100%).

**Table 3.** Factors influencing the total patient experience scores related to physicians in the multiple linear regression.

Variables	B^a^	SE^b^	*t* test	*P* value	Adjusted *P* value^c^
Constant	3.241	0.211	15.35	<.001^d^	
Whether the conversational agent was used (ref: No)					
Yes	0.298	0.076	3.95	<.001^d^	.013
Sex (ref: male)					
Female	0.047	0.082	0.57	.57	.780
Age (ref: 26‐30 y old)					
18‐25	0.197	0.127	1.55	.12	.496
31‐40	−0.111	0.117	−0.95	.34	.678
40	0.189	0.150	1.26	.21	.496
Marital status (ref: unmarried)					
Married	0.279	0.117	2.38	.02^d^	.156
Educational level (ref: high school and below)					
College and undergraduate	0.020	0.120	0.16	.87	.906
Postgraduate and above	−0.101	0.148	−0.68	.497	.780
Monthly income (ref: <US $417.97)					
417.97‐696.48	−0.065	0.111	−0.58	.56	.780
696.62‐1114.45	−0.023	0.102	−0.23	.82	.886
≥1114.59	0.172	0.124	1.40	.16	.496
Current living place (ref: Rural areas)					
Urban areas	0.226	0.103	2.20	.03^d^	.182
Self-rated health status (ref: fair)					
good	0.079	0.090	0.88	.38	.678
Very good	0.520	0.110	4.74	<.001^d^	.013
Specialty services (ref: dermatology)					
Internal medicine	0.096	0.112	0.86	.39	.678
Surgery	0.011	0.128	0.08	.93	.933
Obstetrics and gynecology	−0.062	0.156	−0.40	.69	.781
Orthopedics	−0.231	0.171	−1.35	.18	.496
Traditional Chinese medicine	0.222	0.135	1.65	.10	.496
Otorhinolaryngology	−0.240	0.189	−1.27	.21	.496
Ophthalmology	−0.090	0.202	−0.45	.66	.781
Stomatology	0.172	0.131	1.31	.19	.496
Physician title (ref: senior)					
Deputy Senior	−0.082	0.090	−0.91	.36	.678
Intermediate	0.036	0.089	0.40	.69	.781
Junior	−0.070	0.173	−0.40	.69	.781
Whether this outpatient visit was a re-visit (ref: Yes)					
No	−0.062	0.102	−0.60	.55	.780

a B: unstandardized regression coefficient.

b SE: standard error.

c The adjusted *P* values of independent variables were adjusted by Benjamini-Hochberg procedure.

d Represents the variable is significant in the multiple linear regression.

Among these control factors, self-rated health status (*B*=0.520, *P*=.013) was a significant factor that influenced the total patient experience scores related to physicians. And these residents who rated their health status as “very good” were more likely to report a higher patient experience score related to physicians during this outpatient visit.

Moreover, the regression model explained 25.54% of the variance in the total patient experience scores related to physicians (*R*^2^=0.2554). And we further calculated the value of the variance inflation factor to check for collinearity. The variance inflation factor value of all independent variables was between 1.15 and 2.51, which indicated that there was no collinearity.

## Discussion

### Principal Findings

We found that the use of artificial intelligence-assisted conversational agents averagely increased the total patient experience scores related to physicians by 7.51%. In this study, conversational agent users reported a better experience in the physician-patient communication, access to health information, short-term outcomes, and general satisfaction as well as their specific 19 items.

The use of artificial intelligence-assisted conversational agents can improve communication efficiency between physicians and patients during outpatient visits. After completing the registration, the patients can click on the “pre-consultation” on the registration and appointment page of the hospital’s mobile app by using their mobile phones and then engage in a dialog with artificial intelligence chatbots [14]. And a corresponding structured preconsultation report is formed and delivered to their visit physicians, and the physicians thereby quickly grasp the patients’ conditions before a consultation and further conduct a targeted inquiry [11]. This could improve the consultation efficiency between physicians and patients and, in turn, contribute to positive outcomes, such as making physician-patient communication better, accessing more targeted health information, ameliorating short-term outcomes, and increasing general satisfaction. These positive outcomes that appear to result from the use of the artificial intelligence-assisted conversational agents have been confirmed in our quantitative study.

Currently, the situation where Chinese patients have a lack of adequate communication with their physicians during outpatient visits in the tertiary hospitals has not been effectively improved, which hinders the improvement of the current physician-patient relationship [23,24]. The artificial intelligence-assisted conversational agents can help patients communicate more effectively with their visit physicians and access more targeted health information within the existing limited time. This could, in turn, result in a better physician-patient relationship during outpatient visits.

Our quantitative study found that the overall patient experience related to physicians could be improved significantly when the artificial intelligence-assisted conversational agent was used during outpatient visits. This finding could be supported by the findings of Lu et al [1] regarding the effect of mobile health apps on the patient experience that using mobile health apps could improve the patient experience. And the extent to which the artificial intelligence-assisted conversational agents improved the patient experience in this study was higher than that of the mobile health apps reported in the previous research in 2018 (7.51% vs 5.35%) [1]. This difference might be relevant to the fact that the artificial intelligence-assisted conversational agents not only allow the patients to report their information in detail before a consultation, but also further make them communicate more efficiently with their visit physicians within the existing limited time [11,14], thereby bringing them a better communication experience with their visit physicians. In contrast, the past mobile health apps were dedicated to saving the patients’ waiting time throughout their visits and thereby improving their visit experience [1]. More importantly, there is increasing evidence supporting that improved health care system delivery could improve the patient experience, which in turn brings a better health outcome to patients [3,25]. Therefore, we have reason to believe that the increased application and use of the artificial intelligence-assisted conversational agents in outpatient departments could contribute to a better health outcome for outpatients.

Nevertheless, the current application of artificial intelligence-assisted conversational agents in outpatient departments is mainly in the tertiary public hospitals in big cities of China (eg, Shanghai, Shenzhen, and Wuhan). And the existing survey research in 2022 found that six months after tertiary hospitals in Shanghai deployed the artificial intelligence-assisted conversational agents in outpatient departments, the patients’ usage rates fell short of expectations (26% and 20% for the second- and fourth-ranked hospitals, respectively) [11]. Therefore, we suggest that public hospitals should be encouraged to promote the application of the artificial intelligence-assisted conversational agents in outpatient departments and integrate them into the functions of their existing mobile health apps to continuously improve the patient experience related to physicians during outpatient visits. More importantly, given that public hospitals in less-developed regions generally lack sufficient funds to deploy the artificial intelligence-assisted conversational agents, we also suggest that the Chinese government should increase financial support for these public hospitals. This would accelerate the promotion of the artificial intelligence-assisted conversational agents in outpatient departments and thereby improve the patient experience on a large scale.

Moreover, our study also showed that self-rated health status was a significant factor influencing the patient experience related to physicians during outpatient visits. This result is similar to the findings of several studies on the effect of mobile health apps on the patient experience that the patients who rated their health status better were more likely to report a better patient experience [1]. Another study by Li et al also indicated that the patients with worse self-rated health status would be more likely to experience a negative physician-patient relationship [26]. Therefore, we also suggest that hospitals should make full use of the artificial intelligence-assisted conversational agents to further improve the medical experience of these patients with worse self-rated health status.

### Limitations

There are some limitations in this study. First, data collection was self-reported by adult residents based on their outpatient experience within the past 2 weeks, which might have a recall bias and selection bias. Second, our conclusions might have been biased by distributions, such as sex and age. Therefore, after controlling for the influence of the confounding factors on participant characteristics, multiple linear regression analysis was performed to examine whether the use of artificial intelligence-assisted conversational agents improves the patient experience related to physicians during outpatient visits, which could have resulted in a reliable and stable conclusion. Third, patients can freely choose to use or not use the artificial intelligence-assisted conversational agents during outpatient visits, which might be influenced by these factors (eg, digital literacy, education, and general attitude toward health technology). And this might also act as a confounder in masking the patient experience. Furthermore, further research is necessary to explore the intrinsic mechanism by which the use of artificial intelligence-assisted conversational agents improves the patient experience related to physicians during outpatient visits.

### Conclusions

Our work provides evidence supporting the use of artificial intelligence-assisted conversational agents for improving the patient experience related to physicians during outpatient visits, especially in terms of making physician-patient communication better, accessing more targeted health information, ameliorating short-term outcomes, and increasing general satisfaction. All of these may further bring positive health outcomes to patients. Therefore, we suggest that public hospitals should consider the benefits of the artificial intelligence-assisted conversational agents and actively deploy the conversational agents in outpatient departments so as to continuously improve the patient experience related to physicians during outpatient visits.
